# Supplementary material for: Impaired cerebrovascular reactivity correlates with reduced retinal vessel density in patients with carotid artery stenosis: Cross-sectional, single center study
Source: PLoS One. 2023 Sep 14;18(9):e0291521. doi: 10.1371/journal.pone.0291521 (PMC10501613; doi:10.1371/journal.pone.0291521)
Supplement: S1 Checklist — (DOCX) [file pone.0291521.s001.docx]

STROBE Statement—checklist of items that should be included in reports of observational studies

|  | Item No. | Recommendation | Page  No. | Relevant text from manuscript |
| --- | --- | --- | --- | --- |
| **Title and abstract** | 1 | (*a*) Indicate the study’s design with a commonly used term in the title or the abstract | 3 | “In this cross-sectional study the middle cerebral artery (MCA) blood flow velocity was measured by transcranial Doppler (TCD) and, simultaneously, continuous non-invasive arterial blood pressure measurement was performed on the radial artery by applanation tonometry.” |
|  |  | (*b*) Provide in the abstract an informative and balanced summary of what was done and what was found | 3 | “Results: Twenty-four ICA stenotic patients were evaluated. Both CVR and retinal VD were characterized. There was a significant, negative correlation between CAR-THRR and VDPP_small_ vessel type (p<0.05), as well as between VDPP_all_ vessel types (p<0.05), and also similar correlation between CAR-THRR and VDP-WI_small_ (p<0.05) and between VDP-WI_all_ (p<0.05).” |
| Introduction | | | |  |
| Background/rationale | 2 | Explain the scientific background and rationale for the investigation being reported | 5 | “Most of the total deaths in the countries of the world, regardless of their economic development, is due to cardiovascular diseases, and its most common pathological basis is atherosclerosis. “  “The considerable portion of cerebral ischemic events (~30%) result from atherosclerotic steno-occlusive disease of the internal carotid artery (ICA).”  “These parallel pathological events are logical consequences of the fact that the cerebral and retinal circulations are developmentally, anatomically, and physiologically interconnected.”  “However, there is few if any study investigating simultaneously the cerebral and retinal vascular alterations in patient with ICA atherosclerotic stenosis, which would however show the general presence of atherosclerotic vascular disease and facilitate the correct diagnosis and the consequent treatments. On the basis of the above we hypothesized that in patients with atherosclerotic ICA stenosis – which is likely an indicator of systemic atherosclerotic vascular diseases – affecting both cerebral and retinal circulations and there is a correlation between the functional responses of cerebral vessels.” |
| Objectives | 3 | State specific objectives, including any prespecified hypotheses | 6 | “The aim of the present study was to characterize the parameters indicating cerebrovascular reactivity and retinal vascular density changes and assess if correlation exist between them in older adults with atherosclerotic carotid stenosis.” |
| Methods | | | |  |
| Study design | 4 | Present key elements of study design early in the paper | 6 | “This cross-sectional study was approved by the Regional, Institutional Scientific Research Ethics Committee of Semmelweis University (SE RKEB permit numbers: 256/2018 and 84/2019) and registered on the ClinicalTrials.gov website (Reg No: NCT03840265).” |
| Setting | 5 | Describe the setting, locations, and relevant dates, including periods of recruitment, exposure, follow-up, and data collection | 6 | “The study was conducted according to the guidelines of the Declaration of Helsinki. Patients with significant ICA stenosis were evaluated at the Department of Vascular Surgery and Endovascular Surgery of Semmelweis University after providing them with detailed oral information and gaining their written consent, were consecutively enrolled between 01.01.2019 and 30.09.2021. and data processing began after patient enrollment. Authors participating in data curation, project administration and investigation had access to information that could identify individual participants during or after data collection.” |
| Participants | 6 | (*a*) *Cohort study*—Give the eligibility criteria, and the sources and methods of selection of participants. Describe methods of follow-up  *Case-control study*—Give the eligibility criteria, and the sources and methods of case ascertainment and control selection. Give the rationale for the choice of cases and controls  *Cross-sectional study*—Give the eligibility criteria, and the sources and methods of selection of participants | 6-7 | “Inclusion criteria for the study were threefold: significant carotid artery stenosis (≥ 70%) was determined by 256-slice scanner computer tomography angiography (CTA) (Brilliance iCT 256, Philips Healthcare, Best, Netherlands) based on the North American Symptomatic Carotid Endarterectomy Trial (NASCET) criteria and planned endarterectomy. Exclusion criteria for the TCD examination were atrial fibrillation, extensive embolic plaques in the common carotid artery prone to fragment when compressed, and carotid sinus hyperesthesia. Patients with macular degeneration, glaucoma, vitreomacular diseases, previous intraocular anti-VEGF (vascular endothelial growth factor) injection, clinically significant media opacity, and nystagmus were excluded from OCTA examination. Demographic and medical history data of the patients were prospectively collected.” |
|  |  | (*b*) *Cohort study*—For matched studies, give matching criteria and number of exposed and unexposed  *Case-control study*—For matched studies, give matching criteria and the number of controls per case |  |  |
| Variables | 7 | Clearly define all outcomes, exposures, predictors, potential confounders, and effect modifiers. Give diagnostic criteria, if applicable | 10-12 | 2Parameters of blood flow velocity changes in response to CCC” : paragprahg begins on page 10 row 158 “OCTA variables” : parapgraph begin on page 12, row 194 |
| Data sources/ measurement | 8* | For each variable of interest, give sources of data and details of methods of assessment (measurement). Describe comparability of assessment methods if there is more than one group | *7-12* | “Patients underwent CT angiography as part of standard-of-care diagnostic evaluation using a routine clinical imaging protocol. Patients with significant (>70%), symptomatic (with ipsilateral, hemispheric, non-lacunar infarct or transient ischemic attack in the past 6 months) and asymptomatic (without ipsilateral, hemispherical infarct) ICA stenosis were screened for enrolment.” “Transcranial Doppler (TCD) study protocol The blood flow velocity (BFV) in the MCAs was recorded with a bilateral, fixed TCD transducer (2 MHz, DWL Multi-Dop T2, Sipplingen, Germany) at rest, in semi-sitting position through the transtemporal insonation window at a depth of 45–55 mm. The transducers were adjusted bilaterally using a metallic holder to obtain maximal signal intensity. The analog output of the TCD equipment was the envelope fitted to the maximum of the flow velocity power spectrum after fast-Fourier transformation. During TCD measurements continuous, non-invasive, beat-to-beat arterial blood pressure (ABP) monitoring was performed with radial artery applanation tonometry (Colin-BP508, Hayashi Komaki Aichi, Japan). Calibration to mmHg before and after every common carotid artery compression test was made by the same equipment’s sphygmomanometer.” *“*Data processing Digitization of analog signals was performed in parallel on 3 channels (TCD1, TCD2, ABP tonometry) with a sampling frequency of 500 Hz. Raw data per each patient were stored in European Data Format (EDF) files. The files were imported, digitally filtered, and segmented using the LabChart software (AdInstruments, LabChart ver. 8, Colorado Springs, CO, USA). The pre-processed data segments were stored in separate text files. These files were further processed by a Python code. The program script interpolated the channels linearly into 0.5 second equidistant intervals. The interpolated data was exported to Microsoft Excel and the amplitude and time variables of the vascular reactivity were calculated.” “Optical coherence tomography angiography (OCTA) study protocol OCTA is a fast, non-invasive technique, that allows visualization of blood flow in different layers of the retina and choroid without the need of any contrast agent injection. OCTA measures the movement of red blood cells in the blood vessels, which provides information about the perfusion of the different tissues. In the present study, on the same patients undergoing TCD measurements OCTA examination was performed with the AngioVue OCTA system, using the SSADA (split-spectrum amplitude-decorrelation angiography) software algorithm (RTVue XR Avanti with AngioVue, Optovue Inc, Fremont, CA, USA). (Figure 2.) The software evaluates the image quality (scan quality - SQ) of the OCTA recordings on a 10-point scale, and only test results with an SQ value exceeding 5 points were included in the study. (34) Images containing motion artifacts (such as white line artifacts, vessel discontinuities, vessel doubling, or noise), segmentation errors, or projection artifacts were excluded. (34) Three OCTA examinations of the macular, papillary and peripapillary areas were performed on each patient, and the best image quality was analyzed during data processing.“ |
| Bias | 9 | Describe any efforts to address potential sources of bias | 23 | Limitations The limitation of our study is the low number of patients, primarily due to the missing or very narrow temporal acoustic insonation window in 20-30% of the study group, likely due to the age of and gender of patients. In our study, the rate of occurrence of an inadequate temporal insonation window is higher than in literature data, which we explain by the relatively high average age of the patients and our strict TCD examination protocol. |
| Study size | 10 | Explain how the study size was arrived at | 7 | “During the study period, 89 of 108 patients with significant ICA stenosis agreed to participate in the study. Among these, 33 patients could not be examined with TCD due to a missing or inadequate insonation window, and 32 patients were excluded from the study group due to OCTA exclusion criteria. Finally, we analyzed the TCD and OCTA results of 24 patients with significant ICA stenosis. “ |

Continued on next page

| Quantitative variables | 11 | Explain how quantitative variables were handled in the analyses. If applicable, describe which groupings were chosen and why | 10-11 | “Parameters of blood flow velocity changes in response to CCC Transient hyperemic response ratio (THRR): expresses the change in systolic BFV measured directly after the release of CCC compared to the baseline value.  $\mathbf{THRR}=\frac{V_{S2}{- V}_{S1}}{V_{S1}}$  V_S1_ = average systolic BFV of the baseline; V_S2_ = systolic BFV maximum after the release of the compression. (Figure 1.)  Cerebral arterial resistance - transient hyperemic response ratio (CAR-THRR): cerebral arterial resistance (CAR) refers to reciprocal value of the change in mean BFV_mean_ in MCA (BFV_mean MCA_) per unit change in ABP_mean_  $\mathbf{CAR} = \frac{{ABP}_{mean}}{{BFV}_{mean MCA}}$  CAR cannot be calculated during compression (dashed line on Figure 1/B.), but at baseline and after CCC release. Similar to THRR, CAR-THRR expresses the change in CAR measured at release of CCC compared to the baseline CAR value.  $\mathbf{CAR-THRR=}\frac{\mathrm{CAR}_{2} - \mathrm{CAR}_{1}}{\mathrm{CAR}_{1}}$  Where CAR_1_ = average of baseline CAR; CAR_2_ = CAR at the release of the CCC.  CAR-THRR therefore also takes into account the simultaneous change in arterial blood pressure. If cerebral resistance vessels dilate because of reduced MCA perfusion due to carotid compression, then resistance to flow decreases, hence the value is negative. A less negative value corresponds to a greater decrease in resistance during compression, indicating a greater cerebrovascular reactivity. A lower value for THRR and a higher value for CAR-THRR indicate a decreased cerebrovascular reactivity (CVR).”  “One of the parameters that characterize the retinal vascular network is the vessel density (VD). (30, 35-37) This value expresses the vascularity of the examined area in the skeletonized image. The VD was determined on the papillary area (P) by the software defined 4.5×4.5 mm whole image (whole image - WI) for all vessel types (VDP-WI_all_) and selectively for only the small vessels (VDP-WI_small_). The identification of small vessels is a software-based feature, the pre-determined cut-off for “small” vessels is ˂35 µm, the "all" vessel type measurement displays the vessel density of all vessels regardless of their size. The peripapillary region (PP) was defined by the software as an area enclosed by a 2.5 mm inner ring and a 4.5 mm outer ring, centered on the optic disc. Similarly, on the PP, VD was also determined for all vessel types (VDPP_all_) and selectively for small vessels (VDPP_small_). Another region examined was the superficial (VDM_spf_) and deep (VDM_deep_) vascular network of the macula in a 3×3 mm area. “ |
| --- | --- | --- | --- | --- |
| Statistical methods | 12 | (*a*) Describe all statistical methods, including those used to control for confounding | 12 | “Due to the non-normal distribution of the continuous variables and the relatively small sample size, non-parametric tests were performed: Spearman's correlation test and the Mann-Whitney U test were used. Statistical analysis and data visualization were performed with the Prism Graph Pad software (Version 8.0.1., San Diego, CA USA). P<0.05 was considered as statistically significant.” |
|  |  | (*b*) Describe any methods used to examine subgroups and interactions | 12 | “Due to the non-normal distribution of the continuous variables and the relatively small sample size, non-parametric tests were performed: Spearman's correlation test and the Mann-Whitney U test were used. Statistical analysis and data visualization were performed with the Prism Graph Pad software (Version 8.0.1., San Diego, CA USA). P<0.05 was considered as statistically significant.” |
|  |  | (*c*) Explain how missing data were addressed | 12 | “Missing data were excluded from the calculations.” |
|  |  | (*d*) *Cohort study*—If applicable, explain how loss to follow-up was addressed  *Case-control study*—If applicable, explain how matching of cases and controls was addressed  *Cross-sectional study*—If applicable, describe analytical methods taking account of sampling strategy | 6-7 | “Patients with significant (>70%), symptomatic (with ipsilateral, hemispheric, non-lacunar infarct or transient ischemic attack in the past 6 months) and asymptomatic (without ipsilateral, hemispherical infarct) ICA stenosis were screened for enrolment.”  “Inclusion criteria for the study were threefold: significant carotid artery stenosis (≥ 70%) was determined by 256-slice scanner computer tomography angiography (CTA) (Brilliance iCT 256, Philips Healthcare, Best, Netherlands) based on the North American Symptomatic Carotid Endarterectomy Trial (NASCET) criteria (25) and planned endarterectomy. Exclusion criteria for the TCD examination were atrial fibrillation, extensive embolic plaques in the common carotid artery prone to fragment when compressed, and carotid sinus hyperesthesia. Patients with macular degeneration, glaucoma, vitreomacular diseases, previous intraocular anti-VEGF (vascular endothelial growth factor) injection, clinically significant media opacity, and nystagmus were excluded from OCTA examination. Demographic and medical history data of the patients were prospectively collected.” |
|  |  | (*e*) Describe any sensitivity analyses | NA | NA |
| Results | | | | |
| Participants | 13* | (a) Report numbers of individuals at each stage of study—eg numbers potentially eligible, examined for eligibility, confirmed eligible, included in the study, completing follow-up, and analysed | 7 | “During the study period, 89 of 108 patients with significant ICA stenosis agreed to participate in the study. Among these, TCD examination could not be performed by 18 patients due to a completely missing insonation window, and by further 15 patients TCD evaluation was not possible due to a non-optimal signal-to-noise ratio. 32 patients were excluded from the study group due to OCTA exclusion criteria. Finally, we analyzed the TCD and OCTA results of 24 patients with significant ICA stenosis.” |
|  |  | (b) Give reasons for non-participation at each stage | 7 | “During the study period, 89 of 108 patients with significant ICA stenosis agreed to participate in the study. Among these, TCD examination could not be performed by 18 patients due to a completely missing insonation window, and by further 15 patients TCD evaluation was not possible due to a non-optimal signal-to-noise ratio. 32 patients were excluded from the study group due to OCTA exclusion criteria. Finally, we analyzed the TCD and OCTA results of 24 patients with significant ICA stenosis.” |
|  |  | (c) Consider use of a flow diagram | 7 | written in text |
| Descriptive data | 14* | (a) Give characteristics of study participants (eg demographic, clinical, social) and information on exposures and potential confounders | 13 | Table 1. |
|  |  | (b) Indicate number of participants with missing data for each variable of interest | 12 | Missing data were excluded from the calculations. |
|  |  | (c) *Cohort study*—Summarise follow-up time (eg, average and total amount) | NA | NA |
| Outcome data | 15* | *Cohort study*—Report numbers of outcome events or summary measures over time | NA | NA |
|  |  | *Case-control study—*Report numbers in each exposure category, or summary measures of exposure | NA | NA |
|  |  | *Cross-sectional study—*Report numbers of outcome events or summary measures | *13* | *Table 2.* |
| Main results | 16 | (*a*) Give unadjusted estimates and, if applicable, confounder-adjusted estimates and their precision (eg, 95% confidence interval). Make clear which confounders were adjusted for and why they were included | NA | NA |
|  |  | (*b*) Report category boundaries when continuous variables were categorized | 12 | “OCTA variables One of the parameters that characterize the retinal vascular network is the vessel density (VD). (30, 35-37) This value expresses the vascularity of the examined area in the skeletonized image. The VD was determined on the papillary area (P) by the software defined 4.5×4.5 mm whole image (whole image - WI) for all vessel types (VDP-WI_all_) and selectively for only the small vessels (VDP-WI_small_). The identification of small vessels is a software-based feature, the pre-determined cut-off for “small” vessels is ˂35 µm, the "all" vessel type measurement displays the vessel density of all vessels regardless of their size. The peripapillary region (PP) was defined by the software as an area enclosed by a 2.5 mm inner ring and a 4.5 mm outer ring, centered on the optic disc. Similarly, on the PP, VD was also determined for all vessel types (VDPP_all_) and selectively for small vessels (VDPP_small_). Another region examined was the superficial (VDM_spf_) and deep (VDM_deep_) vascular network of the macula in a 3×3 mm area.” |
|  |  | (*c*) If relevant, consider translating estimates of relative risk into absolute risk for a meaningful time period | NA | NA |

Continued on next page

| Other analyses | 17 | Report other analyses done—eg analyses of subgroups and interactions, and sensitivity analyses | NA | NA |
| --- | --- | --- | --- | --- |
| Discussion | | | | |
| Key results | 18 | Summarise key results with reference to study objectives | 18 | “The salient findings of the present study are that in patients with significant ICA stenosis there were 1) functional deficit of cerebrovascular responsiveness, tested by common carotid artery compression test, 2) severely reduced retinal vessel density and 3) strong correlations between the cerebrovascular functional and retinal vessel density variables. These findings suggest that common alterations and pathomechanisms underlie the atherosclerotic changes in the cerebral and retinal vasculature identified for the first time in ICA stenotic patients by simultaneous use of functional TCD and OCTA modalities. “ |
| Limitations | 19 | Discuss limitations of the study, taking into account sources of potential bias or imprecision. Discuss both direction and magnitude of any potential bias | 23 | “The limitation of our study is the low number of patients, primarily due to the missing or very narrow temporal acoustic insonation window in 20-30% of the study group, likely due to the age of and gender of patients. In our study, the rate of occurrence of an inadequate temporal insonation window is higher than in literature data, which we explain by the relatively high average age of the patients and our strict TCD examination protocol. “ |
| Interpretation | 20 | Give a cautious overall interpretation of results considering objectives, limitations, multiplicity of analyses, results from similar studies, and other relevant evidence | 19-22 | “Hemodynamic basis of reduced cerebrovascular reactivity To test our hypothesis, we have used the non-invasive transcranial Doppler technique (TCD) known to have excellent temporal sensitivity (38-41) to follow the changes in blood flow velocity in the middle cerebral artery (MCA) in various conditions. (39, 42) Assuming that neither the position of the TCD transducer nor the diameter of the examined vessel changes during a cerebral vasoactive stimulus, the change in flow velocity that occurs is proportional to the change in the cerebral blood flow. (43) Previous studies have shown that the detailed analysis of envelope fitted to the maximum of the flow time-frequency (velocity) power spectrum after fast-Fourier transformation (shape, flow direction, average velocity) created from the Doppler frequency shift value is suitable for the analysis and quantification of hemodynamic changes, such as cerebrovascular reactivity (CVR). (28, 40, 44) The response to CCC stimulus can be divided into two phases with changes in opposite directions. The vasodilation induced by manually performed common carotid artery compression evokes considerable hypoperfusion, resulting in an increase in MCA blood flow velocity after cessation of carotid compression, which exceeds the baseline value under intact control. Cerebral arterioles compensate to their maximum vasodilation capacity with maintained pressure autoregulation, and tissue perfusion remains sufficient. This reactive hyperperfusion then results in an opposite process, hyperperfusion-induced vasoconstriction, MCA cerebral blood flow velocity values return to the baseline, the process is probably not passive. (45, 46) When there was an immediate and significant increase in MCA blood flow velocity after cessation of carotid compression, i.e., the THRR was high, vasoconstriction induced by increased flow also occurred rapidly. The absence or delay of transient hyperemia indicates a decrease in reactivity. In previous studies, the index calculated from THRR BFV values proved to be suitable for characterizing reduced cerebral vasoreactivity. (26-29) However, in our correlation analysis, no close relationship was seen with the mentioned BFV index and the OCT values presenting retinal hypoperfusion. When other index that can be determined with the CCC test was used, we found a significant correlation. This index was the CAR-THRR. In the complex evaluation of cerebrovascular reactivity tests, many previous studies emphasize the consideration of arterial blood pressure. (45-48) Thus while the THRR is based only on the BFV change measured in the MCA, the CAR-THRR variable takes into account the simultaneous arterial blood pressure changes in addition to the BFV changes, so the change in cerebral arterial resistance of the MCA after cessation of manually induced (CCC) hypoperfusion, which was used to characterize the cerebral vasoreactivity, can be determined more precisely. Therefore, the CCC test can be considered a dynamic autoregulation testing method, as changes in ABP and BVF are significant within a short time. The resistance calculation of the cerebral vessels is advantageous because it expresses not only the blood flow velocity but also simultaneous arterial blood pressure changes. Therefore, in purpose of CVR characterization, parallel ABP and HR registration is considered essential because these factors are not insignificant when evaluating cerebral vasoreactivity. Normally, the CAR value at the end of CCC test due to maximal vasodilation is low, so the CAR-THRR value is negative. Thus, the decrease in blood flow velocity resistance developed after the cessation of the CCC, which was used to quantify the vasoreactivity of the brain tissue perfused by the MCA, can more accurately characterize the cerebral vascular reserve.  **Role of regional differences in retinal vasculature**  In the present study a correlation was only found between impaired cerebrovascular reactivity and hypoperfusion resulted decreased circulation of papillary and peripapillary regions, but not with the macula region. The difference can be explained by the fact that the anatomical blood supply and regulatory mechanisms of the various intraocular regions are different. (49, 50) The arterial blood supply of the eye is provided by the ICA via several branches of the ophthalmic artery (central retinal artery - ensuring blood supply of the superficial layers of the retina, the short and long posterior ciliary arteries, and the anterior ciliary arteries - mainly suppling the outer retinal layers and eye structures and forming the choroid). (49, 50) In the circulation of the macula, the branches of the central retinal artery are decisive (as known macula normally has an avascular zone at the fovea where these branches are not present). (51) Due to its high metabolic needs of macula, regulation of macular blood flow is controlled primarily by local metabolic mechanisms affecting the central retinal artery branches. (52, 53) In contrast, in the peripapillary circulation the choroid plays an important role. The short posterior ciliary arteries arborize in terminal arterioles to form the arterioles of the dense outer layer of the choroid. (54) Previous studies suggest local metabolic local control does not seem to play a role in choroidal blood flow regulation.  (55, 56) The choroid is richly innervated, and thus neural control mechanisms are the most relevant regulating factors. (57) The papilla is supplied by the peripapillary choroid composed of the scleral short posterior ciliary system and the recurrent choroidal arteries. Regulation, similarly, to the peripapillary region, is controlled by neuronal mechanisms. (58, 59)  Many of the findings of the present study are consistent with previously reported results in the literature. Similarly, to our study, Zhang et al. analyzed the correlation of OCT and TCD procedures in cerebrovascular patients with ischemic and hemorrhagic stroke. They found a significant, positive correlation between baseline MCA blood flow velocity values and retinal vessel density, however, functional TCD test was not used in their study. (60) Bettermann et al. measured in patients with ischemic white matter disease retinal vasoreactivity using a high-frequency flashing light stimulation method and showed that retinal structural microvascular damage is associated with functional impairment, (61) the extent of which correlates with cerebrovascular reactivity based on the TCD-registered breath-holding test, i.e. decreased blood flow (62) velocity response in the MCA. The great advantage of that study was that it combined two functional methods, which are not performed routinely in patients. (61, 63) In line with our findings, a correlation between MCA baseline blood flow velocity parameters and retinal vessel caliber variability was found in patients with intracranial stenosis using a semi-automated computer assisted program (Singapore I Vessel Assessment). (64)  Optical coherence tomography angiography (OCTA) is known to be suitable for providing images of the retinal vascular network with high accuracy. (65-67) The OCTA method is based on the motion-contrast phenomenon, i.e., the position of the moving structures - the red blood cells - constantly changes in the layers of the retinal vascular network, while no such change occurs in poorly perfused or not at all perfused areas. (68) OCTA is a fast and easily reproducible technique, providing qualitative and quantitative results on various regions of the retinal microcirculation based on red blood cells’ movements. (33) The great advantage of the OCTA examination is that it was previously found to be suitable for monitoring retinal circulation changes after ICA revascularization, as several studies reported a significant improvement in retinal circulation after ICA revascularization not only on the ipsilateral but also on the contralateral side. |
| Generalisability | 21 | Discuss the generalisability (external validity) of the study results | 22-23 | Many of the findings of the present study are consistent with previously reported results in the literature. Similarly, to our study, Zhang et al. analyzed the correlation of OCT and TCD procedures in cerebrovascular patients with ischemic and hemorrhagic stroke. They found a significant, positive correlation between baseline MCA blood flow velocity values and retinal vessel density, however, functional TCD test was not used in their study. (60) Bettermann et al. measured in patients with ischemic white matter disease retinal vasoreactivity using a high-frequency flashing light stimulation method and showed that retinal structural microvascular damage is associated with functional impairment, (61) the extent of which correlates with cerebrovascular reactivity based on the TCD-registered breath-holding test, i.e. decreased blood flow (62) velocity response in the MCA. The great advantage of that study was that it combined two functional methods, which are not performed routinely in patients. (61, 63) In line with our findings, a correlation between MCA baseline blood flow velocity parameters and retinal vessel caliber variability was found in patients with intracranial stenosis using a semi-automated computer assisted program (Singapore I Vessel Assessment). (64)  Optical coherence tomography angiography (OCTA) is known to be suitable for providing images of the retinal vascular network with high accuracy. (65-67) The OCTA method is based on the motion-contrast phenomenon, i.e., the position of the moving structures - the red blood cells - constantly changes in the layers of the retinal vascular network, while no such change occurs in poorly perfused or not at all perfused areas. (68) OCTA is a fast and easily reproducible technique, providing qualitative and quantitative results on various regions of the retinal microcirculation based on red blood cells’ movements. (33) The great advantage of the OCTA examination is that it was previously found to be suitable for monitoring retinal circulation changes after ICA revascularization, as several studies reported a significant improvement in retinal circulation after ICA revascularization not only on the ipsilateral but also on the contralateral side. (22, 69-72) |
| Other information | |  | | |
| Funding | 22 | Give the source of funding and the role of the funders for the present study and, if applicable, for the original study on which the present article is based | 24-25 | Role of the funders: financial support.  “The study was supported by the National Office for Research, Development and Innovation (Project no. NKFI-K129277 ("Evaluation of cerebrovascular events in patients with occlusive carotid artery disorders based on morphological and hemodynamic features") has been implemented with the support provided by the Ministry of Innovation and Technology of Hungary from the National Research, Development, and Innovation Fund. NKFI-1 K OTKA 132596 K­_19, TKP2021-EGA-37 of MIT of Hungary-NRDI TKP2021-EGA funding and HAS/MTA Post-Covid 2021-34.” |

*Give information separately for cases and controls in case-control studies and, if applicable, for exposed and unexposed groups in cohort and cross-sectional studies.

**Note:** An Explanation and Elaboration article discusses each checklist item and gives methodological background and published examples of transparent reporting. The STROBE checklist is best used in conjunction with this article (freely available on the Web sites of PLoS Medicine at http://www.plosmedicine.org/, Annals of Internal Medicine at http://www.annals.org/, and Epidemiology at http://www.epidem.com/). Information on the STROBE Initiative is available at www.strobe-statement.org.
