# Supplementary material for: Impaired cerebrovascular reactivity correlates with reduced retinal vessel density in patients with carotid artery stenosis: Cross-sectional, single center study
Source: PLoS One. 2023 Sep 14;18(9):e0291521. doi: 10.1371/journal.pone.0291521 (PMC10501613; doi:10.1371/journal.pone.0291521)
Supplement: S1 Appendix — The text summarizes the technical description of TCD measures. (DOCX) [file pone.0291521.s002.docx]

## Transcranial Doppler (TCD) study protocol

The blood flow velocity (BFV) in the MCAs was recorded with a bilateral, fixed TCD transducer (2 MHz, DWL Multi-Dop T2, Sipplingen, Germany) at rest, in semi-sitting position through the transtemporal insonation window at a depth of 45–55 mm. The transducers were adjusted bilaterally using a metallic holder to obtain maximal signal intensity. The analogue output of the TCD equipment was the envelope fitted to the maximum of the flow velocity power spectrum after fast-Fourier transformation.
