# Supplementary material for: Impaired cerebrovascular reactivity correlates with reduced retinal vessel density in patients with carotid artery stenosis: Cross-sectional, single center study
Source: PLoS One. 2023 Sep 14;18(9):e0291521. doi: 10.1371/journal.pone.0291521 (PMC10501613; doi:10.1371/journal.pone.0291521)
Supplement: S2 Appendix — The text summarizes the software data processing. (DOCX) [file pone.0291521.s003.docx]

## Data processing

Digitization of analog signals was performed in parallel on three channels (TCD1, TCD2, ABP tonometry) with a sampling frequency of 500 Hz. Raw data per each patient were stored in European Data Format (EDF) files. The files were imported, digitally filtered, and segmented using the LabChart software (AdInstruments, LabChart ver. 8, Colorado Springs, CO, USA). The pre-processed data segments were stored in separate text files. These files were further processed by a Python code. The program script interpolated the channels linearly into 0.5 second equidistant intervals. The interpolated data were exported to Microsoft Excel and the amplitude and time variables of the vascular reactivity were calculated.
