# Supplementary material for: Impaired cerebrovascular reactivity correlates with reduced retinal vessel density in patients with carotid artery stenosis: Cross-sectional, single center study
Source: PLoS One. 2023 Sep 14;18(9):e0291521. doi: 10.1371/journal.pone.0291521 (PMC10501613; doi:10.1371/journal.pone.0291521)
Supplement: S3 Appendix — The text summarizes the description of the standardized common carotid artery compression test. (DOCX) [file pone.0291521.s004.docx]

**Common carotid artery compression test – CCC test**

The 10 second average of blood flow velocity data preceding the compression was considered the baseline value. The CCC tests were repeated three times in each patient, with a 2-minute break between CCA compressions. The response with the largest amplitude of BFV in MCA was used for statistical analysis.
