# Supplementary material for: Impaired cerebrovascular reactivity correlates with reduced retinal vessel density in patients with carotid artery stenosis: Cross-sectional, single center study
Source: PLoS One. 2023 Sep 14;18(9):e0291521. doi: 10.1371/journal.pone.0291521 (PMC10501613; doi:10.1371/journal.pone.0291521)
Supplement: S4 Appendix — The text summarizes the description of the software-based small vessel definition. (DOCX) [file pone.0291521.s005.docx]

### OCTA small vessel criteria

The identification of small vessels is a software-based feature, the pre-determined cut-off for “small” vessels is ˂35 µm, the "all" vessels measurement displays the vessel density of all vessels regardless of their size. The built in software of the OCT device automatically differentiates the small vessels. Based on the user manual, vessels smaller than 35 microns are classified in this category.
