# Supplementary material for: Impaired cerebrovascular reactivity correlates with reduced retinal vessel density in patients with carotid artery stenosis: Cross-sectional, single center study
Source: PLoS One. 2023 Sep 14;18(9):e0291521. doi: 10.1371/journal.pone.0291521 (PMC10501613; doi:10.1371/journal.pone.0291521)
Supplement: S1 File — (PDF) [file pone.0291521.s006.pdf]

ID	Age	Density WI	Density PF	Density WI	Density PF	Fovea thick	FAZ	No-flow
2	64	40	43,4	40	43,5	255	0,349	0,631
3	66	42	45,5	45,7	48,1	267	0,208	0,423
4	61	41,3	43,8	46,7	50	260	0,403	0,669
8	77	39,5	43,6	45,2	48,6	238	0,358	0,781
13	70	46,3	49,7	47,6	48,9	259	0,258	0,437
19	68	53	55,5	44,5	45,5	345	0,261	0,23
23	78	45,3	48,6	44,2	46,9	249	0,337	0,553
27	53	49,9	52,7	54,9	56,6	241	0,242	0,443
28	72	47	51	47,9	49,9	217	0,438	0,694
56	72	44,7	48,4	48,6	50	256		0,473
30	73	44	46,9	53,1	55,2	252	0,199	0,426
33	77	43,5	45,3	46,9	48,2	278	0,227	0,422
34	61	45,6	48,6	50,7	51,8	278	0,186	0,442
35	76	37,7	40,3	48,2	51,2	229	0,27	0,674
38	75	46	48,9	44,3	46,1	242	0,23	0,424
40	73	40,6	43,9	54,3	55,3	255	0,176	0,476
42	62	45,6	49,2	46,8	49,7	243	0,357	0,534
48	59	45,5	47,9	55,8	57,4	265	0,143	0,344
49	83	44,4	47,3	48,9	50,5	265	0,306	0,397
50	70	42,9	44,4	49,4	50,3	314	0,137	0,165
52	67	31,7	34,6	43,5	46,9	244	0,458	0,989
53	67	38,2	40,2	49,5	50,4	232	0,268	0,577
54	65	48,4	50,7	51,5	52,9	278	0,259	0,408

IQ	VD RPC Wi	VD RPC WI	VD RPC PP	VD RPC PP	RNFL perip	THRR	CAR-TAHRF	smoking
7	46,3	51,8	48	53,3	91	0,3825	-0,15976	1
8	51,4	56,5	53,4	58,2	97	0,35534	-0,27665	0
8	50,7	55,6	53,3	58,2	100	0,00161	-0,09214	0
7	50,1	57,5	53,4	60,6	108	0,274417	-0,40461	1
8	47,2	52,9	50,3	55,3	97	-0,0165	-0,06587	0
8	50,4	58,2	54,4	61,7	128	0,029023	-0,15547	0
8	49,5	55,5	54,3	60	110	0,142246	-0,23437	0
9	49,8	57,4	52,8	60	123	0,339783	-0,15098	1
8	48,5	55,2	51,1	57,6	95	-0,00536	-0,22207	0
8	49	54,5	51,1	56,9	110	0,24907	-0,18476	0
8	47,5	54,2	47,6	55	100	0,00571	-0,09605	1
8	49,4	57,2	52,4	60,1	131	0,677932	-0,34229	0
9	50,1	56,8	52,4	59,4	114	0,32763	-0,04494	0
6	57,3	57,3	53,9	60,4	117	0,0532	-0,28273	0
8	50,1	55,3	52,9	57,8	113	0,257567	-0,38642	0
9	45,5	52,5	46	53,7	84	0,157384	-0,15694	0
8	48,5	56,5	50,8	58,7	117	-0,06074	-0,00484	1
9	49,6	56	51,4	58	99	0,222432	-0,1819	0
9	47	53,5	48,6	55,6	105	0,14486	-0,00445	0
9	40,7	48,1	44	51,2	97	-0,15078	0,128922	1
6	47,2	52,9	50,7	56	96	0,466612	0,099267	1
8	49,8	56,7	52	58,7	105	0,09386	-0,25147	1
9	50,4	57,5	53	59,8	142	0,313786	-0,21604	0

diabetic	symptomat	contralat	steno-occl
0	1	0	
1	0	0	
0	0	0	
0	0	0	
1	0	0	
1	0	0	
1	0	0	
0	0	0	
0	0	1	
0	0	0	
0	1	0	
0	0	0	
1	0	1	
0	0	0	
0	1	0	
0	0	0	
0	1	0	
0	0	1	
0	0	0	
1	0	1	
0	1	1	
1	1	1	
0	0	1	
