# Supplementary material for: Impaired cerebrovascular reactivity correlates with reduced retinal vessel density in patients with carotid artery stenosis: Cross-sectional, single center study
Source: PLoS One. 2023 Sep 14;18(9):e0291521. doi: 10.1371/journal.pone.0291521 (PMC10501613; doi:10.1371/journal.pone.0291521)
Supplement: S2 File — (PDF) [file pone.0291521.s007.pdf]

SEMMELWEIS EGYETEM
REGIONÁLIS, INTÉZMÉNYI
TUDOMÁNYOS ÉS
KUTATÁSETIKAI BIZOTTSÁG
1091 Budapest, Üllői út 93.
Telefon: 215-5038 Fax: 215-6228
Levélcím: 1450 Budapest, Pf.: 9/41.
e-mail: sotonyi.peter@med.semmelweis-univ.hu
http://semmelweis.hu/rkeb

SEMMELWEIS UNIVERSITY
REGIONAL AND INSTITUTIONAL
COMMITTEE OF SCIENCE
AND RESEARCH ETHICS
Üllői st. 93., Budapest, H-1091
Phone: (36-1) 215-5038 Fax: (36-1) 215-6228
Mailing address: H-1450 Budapest P.O.B. 9/41.

President: Péter Sótonyi MD.
forensic medicine

Secretary: Ágnes Dósa MD
doctor and lawyer

Members:
Tamás Béres MD
theologian

Ildikó Danis MD
mental health professional

György Fekete MD
pediatrician

György Herhuss
engineer, layman

László Imrei MD.
otorhinolaryngologist

Ferenc Juhász MD.
surgeon, health insurance
professional

Lászlóné Kelemen
economist, layman

László Köles MD.
pharmacologist

Tiborné Láng
head nurse of the University

Krisztina Márton MD.
dentist

Attila Molvarec MD
obstetrician gynecologist

István Pulay MD
surgeon

Anikó Somogyi MD.
internist, pharmacologist,
lipidologist

István Takács MD.
internal medicine
endocrinologist,
pharmacologist

Ágnes Zana MD.
ethical professional

Number: 256/2018.
Protocol: -

Péter Sótonyi
Professor
Department of Vascular Surgery

Budapest

Subject: Research entitled "Morphological and hemodynamic characteristics of cerebrovascular events in patients with carotid artery stenosis"

Dear Professor,

The Regional, Institutional Scientific and Research Ethics Committee of Semmelweis University made the following decision at its meeting held on December 18, 2018:

The committee found the research plan appropriate from a professional and ethical point of view, as well as the material and personnel conditions of the institution suitable for conducting the research.

This decision of the committee was made on the basis of the Hungarian Health Act of 1997. (CLIV) and the Ministry of Health Decree on medical research conducted on humans.

We would like to draw your attention to strict compliance with data protection legislation. (Hungarian Health Act of 1997. XLVII. § 21: on the management and protection of health and related personal data. Hungarian Health Act of 2011. CXII.: on the right to self-determination of information and freedom of information.)

Furthermore, we would like to inform you that during the conduct of the research, investigation or clinical trial the provisions of the GDPR (REGULATION OF THE EUROPEAN PARLIAMENT AND OF THE COUNCIL (EU) 2016/679 *(April 27, 2016) on the protection of natural persons with regard to the processing of personal data and on the free flow of such data and on the repeal of Regulation (95/46/EC)* **must be taken into account and its provisions must be observed.**

For your information, the Regional, Institutional Scientific and Research Ethics Committee of Semmelweis University can check at any time whether the research and examination are carried out in accordance with the provisions of the research plan and the license. (Hungarian Ministry of Health Decree 23/2001 (V.9) § 18)

After completion of the investigation, the report must be sent to the Committee.

Budapest, January 14, 2019.

Semmelweis Egyetem
Regionális, Intézményi
Tudományos és Kutatásetikai Bizottság
1091 Budapest, Üllői út 93.
Telefon: 215-5038 Fax: 215-6228
Levélcím: 1450 Budapest, Pf: 9/41

Ágnes Dósa MD
Secretary
